# Development of a single-session physiotherapy and self-management intervention for the treatment of primary traumatic anterior shoulder dislocation for the ‘*Acute Rehabilitation following Traumatic anterior shoulder dISlocAtioN (ARTISAN)’* multi centre RCT

**DOI:** 10.1016/j.physio.2021.06.002

**Published:** 2021-12

**Authors:** ZiHeng Liew, Bruno Mazuquin, David R. Ellard, Eleni Karasouli, Stephen Drew, Chetan Modi, Howard Bush, Martin Underwood, Rebecca S. Kearney

**Affiliations:** a University of Warwick, Clinical Trials Unit, Gibbet Hill Road, Coventry, CV4 7AL, United Kingdom; b Manchester Metropolitan University, Manchester, M15 6BH, United Kingdom; c University Hospitals Coventry & Warwickshire NHS Trust, Trauma and Orthopaedics, Clifford Bridge Road, Coventry, CV2 2DX, United Kingdom

**Keywords:** Shoulder dislocation, Rehabilitation, Physiotherapy

## Abstract

**Objective:**

Optimum physiotherapy management for people with a conservatively managed primary traumatic anterior shoulder dislocation is not known. The purpose of the ARTISAN trial is to compare the clinical and cost-effectiveness of a course of usual care physiotherapy with a single session of physiotherapy and self-management, the ARTISAN intervention. ARTISAN is a UK multi-centre, two-arm, parallel group, randomised controlled trial with 1:1 treatment allocation.

**Design:**

The intervention was developed following the Medical Research Council framework for developing and evaluating complex interventions and will be reported in line with the template for intervention description and replication checklist (TIDieR) and the Consensus on Exercise Reporting Template (CERT). It was informed by published research, national clinical guidelines, current clinical practice and patient and public involvement.

**Results:**

The ARTISAN intervention comprises education (Phase 1), progressive exercise (Phase 2 and Phase 3) and an optional return to sport component (Phase 4). Behaviour change strategies are embedded throughout intervention. The single session of physiotherapy is delivered by a chartered physiotherapist, within the first six weeks of injury, in an NHS outpatient setting. At the end of the initial session, paper-based booklets and/or a patient website with the same content are provided to participants to aid self-management and progression though the four phases of the trial intervention.

**Conclusion:**

The ARTISAN intervention was successfully implemented throughout the internal pilot and is suitable for testing in the subsequent definitive RCT ARTISAN trial.

Trial Registration Number ISRCTN63184243

## Introduction

The shoulder is the most frequently dislocated joint [1], [2], [3]. Incidence is reported to be 8.2–23.9 per 100,000 people per year, 97% of these are anterior dislocations [4], [5], [6]. Traumatic Anterior Shoulder Dislocation (TASD) occurs when the humeral head is displaced out of the glenoid fossa because of an excessive force. A UK cohort study found that men aged 16–20 years and women aged 61–70 years have the highest incidences of TASD [7]. This bimodal distribution is similarly reported for US, Canada and Denmark [6], [8], [9].

After a primary TASD people experience pain, functional limitation and are at high risk of future dislocations [1], [2], [10]. A course of physiotherapy aims to restore a painless and functional shoulder by using exercises to retrain muscles to maintain stability, restore joint movement and reduce the risk of re-dislocation [11]. A Cochrane review found no evidence that physiotherapy achieved these aims [3]; Dutch national guidelines explicitly state not to refer to physiotherapy after a TASD [1] and UK guidelines cite referral *‘may be helpful’* [2].

‘*The Acute Rehabilitation following Traumatic anterior shoulder dISlocAtioN’* (ARTISAN) trial was funded by the National Institute of Health Research Health Technology Assessment Programme (NIHR HTA 16/167/56) in response to the lack of high-quality evidence and clinical consensus for TASD management. The authors describe here the development and implementation the ARTISAN trial intervention.

## ARTISAN trial overview

ARTISAN is a UK multi-centre, two-arm, parallel group, randomised controlled trial with 1:1 treatment allocation. The purpose of the study is to find out if a course of usual physiotherapy is clinically and cost effective when compared to a single session of physiotherapy with self-management materials [12].

Study recruitment is ongoing across UK NHS services and will continue until 478 participants are randomised. People aged 18 years or over and have a primary TASD confirmed radiologically are eligible for the study. All baseline data are collected prior to randomisation including any new or pre-existing injuries in addition to the TASD.

People with bilateral shoulder dislocations, having first line surgical treatment, unable to receive physiotherapy within six weeks, or have significant neurovascular complication are excluded from the study.

All participants receive an initial physiotherapy appointment with a chartered physiotherapist, in an UK outpatient setting. After participants have received the initial session, the ARTISAN trial intervention, they are then randomised to either 1) the initial ARTISAN trial intervention session only with self-management materials or 2) the offer of further physiotherapy delivered according to participating sites’ usual practice.

## Developing the ARTISAN trial intervention

**Fig. 1:**
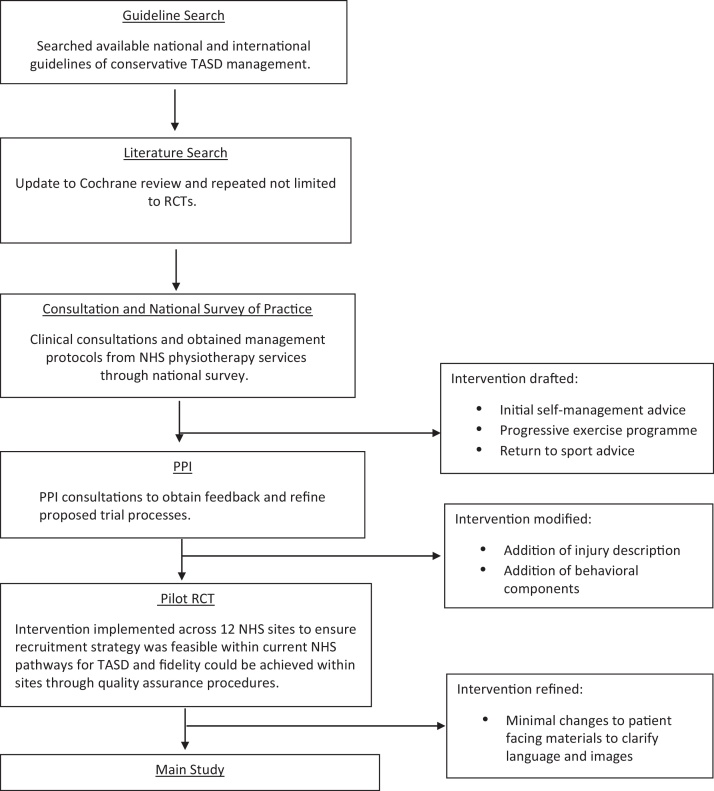
ARTISAN intervention development summary flowchart.

The ARTISAN trial intervention was developed following the Medical Research Council (MRC) guidance for developing and evaluating complex interventions [15]. Using an iterative process based on research evidence, clinical guidelines, current practice, clinicians’ and patients’ opinions (Fig. 1), the authors developed a rehabilitation intervention following a TASD injury. The intervention had four phases: 1) education, 2) range of movement exercises 3) strengthening exercises 4) returning to sports. The authors developed an intervention manual for physiotherapists and patient materials consisting of paper booklets and web-based materials.

Our aim was to ensure that the single session Artisan intervention was scientifically grounded, acceptable to patients and clinicians, and deliverable in the UK NHS setting.

### Clinical guidelines

In the UK, the British Elbow and Shoulder Society (BESS) and the British Orthopaedic Association (BOA) published joint guidelines, advocating conservative management for TASD for those aged 25 years and over, alongside early referral to physiotherapy [2]. They did not make any recommendations regarding the content of rehabilitation. Outside of the UK, only one further set of national guidelines were identified. Dutch Orthopaedic Association guidelines stated that physiotherapy is not recommended after a TASD [1].

### Literature review

The authors searched the literature for further studies published after the updated the Cochrane review entitled ‘*Conservative management following closed reduction of traumatic anterior dislocation of the shoulder’* [3]. Using the same search strategies, the authors found no further published RCTs. Since starting ARTISAN an updated Cochrane review was published 2019, which has still found no further RCTs beyond immobilisation methods. The authors performed a second search by not limiting to RCTs, but excluding case studies, which resulted in two case series describing rehabilitation protocols [16], [17].

In parallel to this, the authors obtained full papers included in a second Cochrane review entitled ‘*Surgical versus non-surgical treatment for acute anterior shoulder dislocation*’ [18]. The aim was to collate and summarise rehabilitation protocols following conservatively managed TASD from these RCTs. However, rehabilitation protocols were either absent from the research papers or not sufficiently detailed to replicate.

Within the limited literature identified, there was a consensus on a phased approach to rehabilitation based on the underlying mechanism of injury and recovery time scales; beginning with simple range of movement exercises and progressing to strengthening exercises that are manipulated to be easier or more challenging by altering load, frequency and repetitions.

### Consultation and national survey of practice

A synthesis of clinical guidelines and current evidence was used as a basis for consultation exercises at five physiotherapy departments. The findings from these were used to inform a national survey, administered to 43 NHS sites, who had expressed an interest in taking part in the ARTISAN RCT, to establish a) what protocols are in use across the UK and b) current care pathways. Of the 43 responses received prior to piloting the intervention, seven used locally developed physiotherapy protocols. Sites were consistent with an educational component and phased exercise approach for rehabilitation.

### Patient and public involvement (PPI)

The authors presented the intervention to a PPI focus group who framed the intervention around their experiences and expectations of physiotherapy after TASD. The PPI group discussed that although the content was relevant it lacked information to help them understand their injury more fully and aid with adherence to the programme, which the group all agreed was difficult, at times.

Subsequently the intervention was further refined to include behavioural components to facilitate self-management and aid with adherence. This included additional information to improve understanding of the injury and expected length of recovery, goal setting and an exercise log. Following these refinements, the intervention was presented back to both PPI and clinicians for final feedback prior to clinical implementation.

### Internal pilot

An internal pilot was completed November 2018–April 2019 across twelve NHS sites, who randomised 43 participants over this period. Minor refinements were made to patient facing materials to clarify language and images. No changes to the physiotherapy manuals or training materials were required. All trial materials were followed through to the main phase of the study.

### Quality assurance

The trial team implemented a standardised approach of evaluating fidelity [19]. This included biannual direct observations and audio recordings; and self-reporting of the trial intervention delivery. All are checked against a standardised list of items. Any issues identified were discussed by the trial management group, who recommend appropriate action. All clinician’s involved receive timely feedback and any identified training needs are addressed.

### Single session of physiotherapy with self-management materials: ARTISAN intervention

The intervention developed is reported based on the template for intervention description and replication checklist (TIDieR) [20] and the Consensus on Exercise Reporting Template (CERT) [21]. Table 1 provides an overview of the ARTISAN intervention.

**Table 1:** Overview of the ARTISAN intervention as per the TIDieR criteria.

TIDieR criteria	Description	
Brief name	ARTISAN (Acute Rehabilitation following Traumatic anterior shoulder dISlocAtioN)	
Why	Referral to a course of physiotherapy is a common conservative management for TASD. However, the evidence is lacking and there are conflicting clinical guidelines.	
What	The ARTISAN intervention comprises of a standardised, single session up to an hour long with self-management materials. All participants in the study receive this session.	
Materials: participant	• Phase 1 booklet titled ‘Your recovery begins here’ • Phase 2/3 booklet titled ‘Your ARTISAN exercise program’ • Phase 4 booklet titled ‘Completing your recovery’	
	Website with animated videos covering contents based on the Phase 1 to 4 booklets. Also contains an online goal setting page and exercise log. Website is password protected and participants can obtain the password from the booklets	
Materials: physiotherapist	Training: Face-to-face training of the ARTISAN intervention conducted by the ARTISAN trained physiotherapist. Sessions are up to 2 hours long in duration.	
	Therapist manual: detailing all components of the study and the study intervention. Also contains a list of coded exercises as a reference for the online additional physiotherapy form.	
	Post-injury questionnaire: Contains the inclusion/exclusion criteria re-validation checklist, OSIS, QuickDASH, EQ-5D-5L, randomisation form and quality assurance check form.	
Procedure: single physiotherapy session (ARTISAN intervention)	At the single physiotherapy appointment the physiotherapists will: - Re-check eligibility of participants. - Provide the OSIS, QuickDASH, EQ-5D-5L for participant to complete - Conduct an initial assessment - Conduct the ARTISAN advice session as outlined in the Phase 1 booklet.	
	Topics included are: a. What has happened to me? b. What can go wrong? c. How do I stop this happening again? d. How long do I have to wear my sling? e. Should I move my arm? f. How do I control my pain? g. When can I return to usual activities? h. What if something goes wrong? i. Points of contact if complications occur or expected recovery times are not achieved	
	- Provide a progressive exercise plan as outlined in the Phase 2/3 booklet. - Provide strategies to enhance participants’ self-management through goal setting and exercise planning. The Phase 2/3 booklets have sample templates for goal setting and exercise logs. - Discuss strategies for returning to sports if appropriate to participants using the Phase 4 booklet. - Complete the randomisation form and randomise participants either online or via the telephone. - Notify Participants in terms of group allocation based on the results of randomisation. - Complete the quality assurance check form.	
Procedure: Group allocation	**ARTISAN session only:** Participants receive the single physiotherapy (ARTISAN) session. Discharge from physiotherapy. Participants can contact their GP/Orthopaedic team if recovery is not as expected.	**ARTISAN session with follow-up:** Participants receive the initial physiotherapy session and follow-up physiotherapy sessions within four months post-randomisation. The frequency, duration and content of the follow-up sessions are based on the discretion of the treating physiotherapist, as per normal physiotherapy follow-up sessions. The physiotherapists records contents of each follow-up sessions in the additional physiotherapy online form.
Who provides	Physiotherapist working within an existing NHS musculoskeletal service in the UK	
	ARTISAN does not exclude any physiotherapist based on the number of years qualified or experience in treating shoulder conditions.	
How	Face-to-face, virtual or telephone delivered session	
Where	The ARTISAN session is delivered in a UK NHS physiotherapy outpatient setting. For physiotherapists who work as part of the orthopaedic team the session is delivered within a UK NHS orthopaedic clinic setting.	
When and how much	The initial physiotherapy session is delivered within 6 weeks post injury. The session is up to 1 hour long.	
Tailoring	To standardise the sessions across all recruiting sites all physiotherapists deliver the same set of advice, exercises and their progressions. Physiotherapist can tailor the progression of exercises based on participants’ ability during the initial appointment. The repetitions for each exercise and goals set are tailored based on participant’s ability at initial appointment.	
Modification	Minor language and image clarifications to patient facing booklets were made prior to the main phase.	
Intervention fidelity	Monitored centrally via the quality assurance check form and the quality assurance checks conducted by a member of the research team, external to the site research team. If sites are found to deviate from the standards required from the protocol further training either via face-to-face or through the phone are arranged by the study team.	

At each NHS site a physiotherapy member of the research team provides training to the physiotherapists participating at site initiation visits for the delivery of the ARTISAN intervention. The training, lasting up to two hours, includes study overview, trial processes, how to deliver each phase of the ARTISAN intervention and how to use patient materials to support behavioural techniques to improve self-management and adherence.

All aspects of the study intervention are summarised in a physiotherapy manual provided to all sites. The ‘train the trainer’ model is utilised whereby physiotherapists who attend the training sessions cascade the study information to physiotherapists who are unable to attend the training session or start delivering the study intervention at a later time. This is supported by the research team where required.

### Phase 1: initial advice

All participants receive a period of initial immobilisation as per UK national guidelines, for a duration of up to two weeks from date of injury and receive a referral to physiotherapy services [2].

At this time participants are provided with a web-link to Phase 1 of the ARTISAN advice materials and/or provided with a paper based booklet version covering:

• What has happened to me?
• What can go wrong?
• How do I stop this happening again?
• How long do I have to wear my sling?
• Should I move my arm?
• How do I control my pain?
• When can I return to usual activities?
• What if something goes wrong?

### Phase 2: range of movement exercises

All participants then receive a single session of physiotherapy that lasts up to one hour and is administered by an ARTISAN trained physiotherapist. Following routine assessment, the physiotherapist delivers the core set of intervention components as described in Phase 1 in addition to:

• Points of contact if complications occur or expected recovery times are not achieved.
• A core set of progressive Phase 2 range of movement exercises and what they aim to achieve.
• Enhancing self-management behaviours through the addition of goal setting, exercise planning and diaries.

Phase 2 comprises of three shoulder range of motion exercises: active shoulder flexion, active shoulder scaption (lifting the arms from the sides in a slightly forward alignment) and active external rotation. If participants are unable to achieve active shoulder movement there is an option to regress the exercises to active assisted movement using the un-injured arm. The aim of this phase is to introduce gentle shoulder movements to the injured shoulder, improving range and decreasing fear of movement. The authors encourage early movement of the shoulder, in line with UK guidelines [22].

The treating physiotherapists can tailor the starting point of the exercises based on the presentation of the participants during the initial session. For example, if a participant presents with full, pain free, shoulder range of movement in all directions during assessment then they can start their rehabilitation programme in Phase 3.

At the end of the session the physiotherapist provides details of web-based materials, which include all the delivered components in written and video format. Participants are also directed towards web-based materials to support future Phase 3 progressive strengthening exercises, what they aim to achieve and later stage Phase 4 progression to aid return to sports. Participants are offered paper-based alternatives.

### Phase 3: shoulder strengthening exercises

Following the initial physiotherapy session, participants can progress to Phase 3 as pain allows. The main aim of phase three is to progress from range of movement exercises to a progressive set of shoulder strengthening exercises, particularly the rotator cuff as one of the main stabilisers of the shoulder. The authors recommended a repetition range and the frequency to be performed per week but encourage participants to set their own personal goals related to what is achievable to them. For example, the authors suggest performing each exercise in sets of ten repetitions, three times daily, but to amend according to their pain and what is achievable. To monitor progress, participants are provided with an exercise log to record progression over time.

The first series of exercises are isometric shoulder flexion, extension, abduction, adduction, external rotation and internal rotation. The second series of exercises are isotonic active flexion, abduction and external rotation using a light weight. Pain inhibits the activation of the rotator cuff muscles which are the main stabilisers of the shoulder joint [23]. Therefore, isometric exercises could be effective in engaging the rotator cuff muscles without increasing pain due to movement during the early stage of rehabilitation. In addition, the authors have chosen simple isotonic shoulder exercises (i.e.: shoulder flexion/abduction in standing using weights) as our progression for shoulder strength as they are simple to adopt by participants and are functional movements [24].

### Phase 4: returning to sport

This phase is optional for participants who intend to go back to sports. As part of the ARTISAN intervention no specific exercises are advised in this phase. Phase 4 consists of later stage information on how to return to sports, applicable to all levels. Advice includes phasing back into sports via gradual introduction to more contact and overhead activities, through to full training sessions and finally a phased return to a competitive environment (i.e. doing only part of a match to begin with). Participants are also provided with the typical time frame for returning to sports (within six months).

### Behavioural component for self-management and exercise adherence

A behavioural component with techniques to facilitate self-management and enhance exercise adherence is incorporated throughout the ARTISAN intervention. These are based on clinical and patient consultations, and relevant research evidence. The Behaviour Change Technique Taxonomy v1 (BCTv1) was used as a framework [25]. The BCTs are described below:

• *Shaping knowledge & natural consequences:* Participants are provided with information to improve understanding of the condition and its management. Any maladaptive beliefs such as fear avoidance in movement resulting from excessive pain are discussed to counter any participant misconceptions and to give generic advice for pain management.
• *Goal setting & action planning:* Goals could be short term, such as the exact time of the day for the exercises to be performed or the repetition of exercises and frequency to be performed in a week. Goals could also be long term, such as to be able to perform a specific task by a specific time period. Goals should be specific, measurable, achievable, relevant and time-bounded (SMART).
• *Self-monitoring:* Participants are encouraged to utilise the exercise log provided in the booklets to document their rehabilitation progress.

## ARTISAN intervention plus usual physiotherapy

Participants randomised to this group receive the ARTISAN intervention and the offer of further follow up with the physiotherapist. The frequency, duration and the content of the follow-up sessions are based on the discretion of the treating physiotherapist, as per normal physiotherapy follow-up. Data regarding to the exercise prescribed or intervention performed by the physiotherapists for each follow-up sessions are collected using a self-reported online form.

Examples of additional physiotherapy interventions could include plyometric and power-based activity specific strengthening and exercises to promote joint position sense and proprioception. These are not included in the ARTISAN only intervention and can be added at the discretion of the treating physiotherapist for participants randomised to this group.

## Conclusion

This paper outlines the development and details of the ARTISAN intervention for participants following a conservatively managed TASD. The final outcomes will be reported at the end of the ARTISAN trial, leading to direct patient benefit.

**Contribution of paper**

• Summarizes current practice, clinical guidelines and research to date after a primary traumatic anterior shoulder dislocation.
• Describes the development of a single session of physiotherapy and self-management intervention after this injury.

  *Ethical approval*: National Research Ethic Committee approved Ref No.18/WA/0236, 26/07/18). Registered on the International Standard Randomised Controlled Trial Number registry (ISRCTN63184243), 07/09/18. The first site opened to recruitment 05/11/18.

  *Funding*: This trial was funded by 10.13039/501100012618NIHR HTA (16/167/56), 01/06/18. The funder has not been involved in the design of the study. The views expressed are those of the authors and not necessarily those of NIHR or the Department of Health and Social Care.

  *Conflict of interest*: RSK is a member of the UK NIHR HTA CET board, NIHR ICA Doctoral panel and previous member of the NIHR RfPB board. RSK, DE, MU, SD, EK, HB and CM have all been awarded current and previous NIHR research grants. ZL and BM have none to declare. MU and RK are co-investigators on NIHR funded trials receiving additional support from Stryker.

MU has received travel expenses for speaking at conferences from the professional organisations hosting the conferences. He is a director and shareholder of Clinvivo Ltd that provides electronic data collection for health services research. He is part of an academic partnership with Serco Ltd. He was an editor of the NIHR journal series for which he received a fee.

## References

[1] Berendes T.D., Pilot P., Nagels J., Vochteloo A.J., Nelissen R.G. (2015). Survey on the management of acute first-time anterior shoulder dislocation amongst Dutch public hospitals. Arch Orthop Trauma Surg.

[2] Brownson P., Donaldson O., Fox M., Rees Jl, Rangan A., Jaggi A. (2015). BESS/BOA patient care pathways: traumatic anterior shoulder instability. Shoulder Elbow.

[3] Hanchard N.C., Goodchild L.M., Kottam L. (2014). Conservative management following closed reduction of traumatic anterior dislocation of the shoulder. Cochrane Database Syst Rev.

[4] Kroner K., Lind T., Jensen J. (1989). The epidemiology of shoulder dislocations. Arch Orthop Trauma Surg.

[5] Simonet W.T., Melton L.J., Cofield R.H., Ilstrup D.M. (1984). Incidence of anterior shoulder dislocation in Olmsted County, Minnesota. Clin Orthop Relat Res.

[6] Zacchilli M.A., Owens B.D. (2010). Epidemiology of shoulder dislocations presenting to emergency departments in the United States. J Bone Joint Surg Am.

[7] Shah A., Judge A., Delmestri A., Edwards K., Arden N.K., Prieto-Alhambra D. (2017). Incidence of shoulder dislocations in the UK, 1995–2015: a population-based cohort study. BMJ Open.

[8] Leroux T., Wasserstein D., Veillette C., Khoshbin A., Henry P., Chahal J. (2014). Epidemiology of primary anterior shoulder dislocation requiring closed reduction in Ontario, Canada. Am J Sports Med.

[9] Liavaag S., Svenningsen S., Reikeras O., Enger M., Fjalestad T., Pripp A.H. (2011). The epidemiology of shoulder dislocations in Oslo. Scand J Med Sci Sports.

[10] Calandra J.J., Baker C.L., Uribe J. (1989). The incidence of Hill-Sachs lesions in initial anterior shoulder dislocations. Arthroscopy.

[11] Gummesson C., Ward M.M., Atroshi I. (2006). The shortened disabilities of the arm, shoulder and hand questionnaire (QuickDASH): validity and reliability based on responses within the full-length DASH. BMC Musculoskelet Disord.

[12] NIHR (2020). https://fundingawards.nihr.ac.uk/award/16/167/56.

[15] Craig P., Dieppe P., Macintyre S., Michie S., Nazareth I., Petticrew M. (2008). Developing and evaluating complex interventions: the new Medical Research Council guidance. BMJ.

[16] Riccio I., de Sire A., Latte C., Pascarella F., Gimigliano F. (2015). Conservative treatment of traumatic shoulder instability: a case series study. Musculoskelet Surg.

[17] Shin S.J., Yun Y.H., Kim D.J., Yoo J.D. (2012). Treatment of traumatic anterior shoulder dislocation in patients older than 60 years. Am J Sports Med.

[18] Handoll H.H., Almaiyah M.A., Rangan A. (2004). Surgical versus non-surgical treatment for acute anterior shoulder dislocation. Cochrane Database Syst Rev.

[19] Mars T., Ellard D., Carnes D., Homer K., Underwood M., Taylor S.J. (2013). Fidelity in complex behaviour change interventions: a standardised approach to evaluate intervention integrity. BMJ Open.

[20] Hoffmann T.C., Glasziou P.P., Boutron I., Milne R., Perera R., Moher D. (2014). Better reporting of interventions: template for intervention description and replication (TIDieR) checklist and guide. BMJ.

[21] Jack K., McLean S.M., Moffett J.K., Gardiner E. (2010). Barriers to treatment adherence in physiotherapy outpatient clinics: a systematic review. Man Ther.

[22] Ouegnin A., Valdes K. (2020). Client preferences and perceptions regarding a written home exercise program or video self-modeling: a cross-sectional study. J Hand Ther.

[23] Stackhouse S.K., Eisennagel A., Eisennagel J., Lenker H., Sweitzer B.A., McClure P.W. (2013). Experimental pain inhibits infraspinatus activation during isometric external rotation. J Shoulder Elbow Surg.

[24] Edwards P.K., Ebert J.R., Littlewood C., Ackland T., Wang A. (2017). A systematic review of electromyography studies in normal shoulders to inform postoperative rehabilitation following rotator cuff repair. J Orthopaedic Sports Phys Therapy.

[25] Michie S., Richardson M., Johnston M., Abraham C., Francis J., Hardeman W. (2013). The behavior change technique taxonomy (v1) of 93 hierarchically clustered techniques: building an international consensus for the reporting of behavior change interventions. Ann Behav Med.

